# An organized approach to imaging of benign cecal pathologies

**DOI:** 10.1259/bjro.20190043

**Published:** 2020-01-10

**Authors:** Ariel Lewis, Shavitri Mahendiran, Jay Hochsztein, Daniel Alterman, Mark Guelfguat

**Affiliations:** 1 Albert Einstein College of Medicine 1300 Morris Park Avenue Bronx, NY 10461, United States; 2 Department of Radiology, Jacobi Medical Center, 1400 Pelham Parkway South Bronx, NY 10461, United States; 3 Department of Radiology, University of Maryland Medical Center, 22 S Greene Street, Baltimore, MD 21201, United States

## Abstract

Unique problems in cecal embryogenesis and cecal pathology can result in characteristic imaging findings. Familiarity with these findings and utilization of an organized approach help to define the cecum’s role in acute abdominal symptoms. Clinical symptoms associated with cecal diseases can be diverse and misleading. This pictorial essay should provide a framework for an understanding of anatomical, infectious, and inflammatory cecal diseases. Knowledge of a broad spectrum of cecal pathologies contributing to these disorders and their corresponding imaging findings can help a radiologist define the diagnosis and guide proper management.

## Introduction

There are a wide variety of maladies that can affect the cecum. These include: (1) anatomic variants such as cecal malrotation, cecal volvulus, cecal bascule and ileocecal intussusception; and (2) inflammatory and infectious entities such as ulcerative colitis (UC), Crohn’s disease, appendicitis, cecal diverticulitis, clostridium difficile colitis, cytomegalovirus colitis, and tuberculosis. Characteristic features help to distinguish one entity from another. However, there are overlapping features that can make it difficult to make an accurate diagnosis. The purpose of this paper is to provide an organized approach, enabling accurate interpretation of imaging findings. This paper will also discuss the unique role of cecal development and its various pathologies with pictorial representations.

## Embryology

The cecum arises from the last of the six primary intestinal loops that come off the proximal segment of the midgut between the 6th and 10th week of gestation. The cecum looks like a bud and serves as a border between the colon and the ileum. Initially, the cecum can be found in the right upper quadrant. Later, as the midgut rotates, the cecum grows caudally and lies in the right iliac fossa.^1^

### Anatomic variants

#### Cecal malrotation

Cecal malrotation is a malposition of the bowel caused by abnormal rotation of the midgut during embryologic development. While usually asymptomatic, malrotation cannot be ignored due to the potential development of complications like internal hernia and midgut volvulus. Internal hernia is caused by the abnormal positioning of the bowel secondary to the peritoneal fibrous bands of Ladd which attach the cecum to the retroperitoneum in the right lower quadrant. Midgut volvulus is caused by the narrowing of the wide mesenteric attachment.^2^ These complications can present as acute small bowel obstruction or chronic abdominal pain.^3^

**Figure 1.**
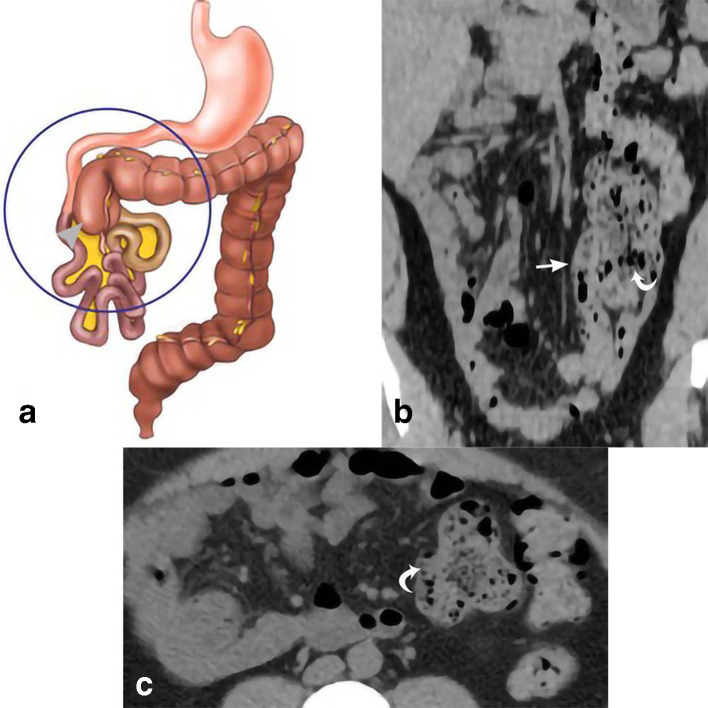
Cecal malrotation. 39-year-old female presents with abdominal pain, right flank pain, and diarrhea. The drawing (a) depicts the classic appearance of malrotation, with the cecum deviated from its normal position in the right lower quadrant to the right upper abdomen (gray arrow head) as a result of a congenital anomaly of rotation. Coronal MPR and axial CT images in our patient (b, c) demonstrate the cecum in the left hemiabdomen (curved white arrows). The position of the terminal ileum (straight white arrow) is to the right of the cecum. MPR, multiplanar reconstruction.

On routine radiography, malrotation can present as a lack of the expected fecal-containing colon in the right hemiabdomen simultaneously occurring with right-sided jejunal markings. However, this is neither sensitive nor specific. On upper gastrointestinal studies, the characteristic finding is absence of the duodenal–jejunal junction in the left parasagittal position. Knowledge of the CT appearance is important because currently CT is routinely performed for a wide range of abdominal complaints. CT can show intestinal malpositioning along with typical retroperitoneal features of the altered anatomic relationship between the superior mesenteric artery and vein and even pancreatic hypoplasia(Figure 1). Knowledge of the typical retroperitoneal abnormalities allows a radiologist to detect clinically silent cases. While contrast enema is considered the gold-standard in finding malrotation, this can be missed due to the presence of a normal cecum in 20% of cases.^2,4^

#### Cecal volvulus

Cecal volvulus is a type of closed-loop obstruction caused by the rotation of the bowel around its mesentery. In order for this to occur, there must be a developmental failure of peritoneal fixation causing the proximal colon to be mobile. Presence of a rotation center, commonly an adhesion, serves to complete the twist of a bowel loop.^5^ Since cecal volvulus may be clinically indistinguishable from uncomplicated small bowel obstruction, early radiologic imaging is important.^6^

**Figure 2.**
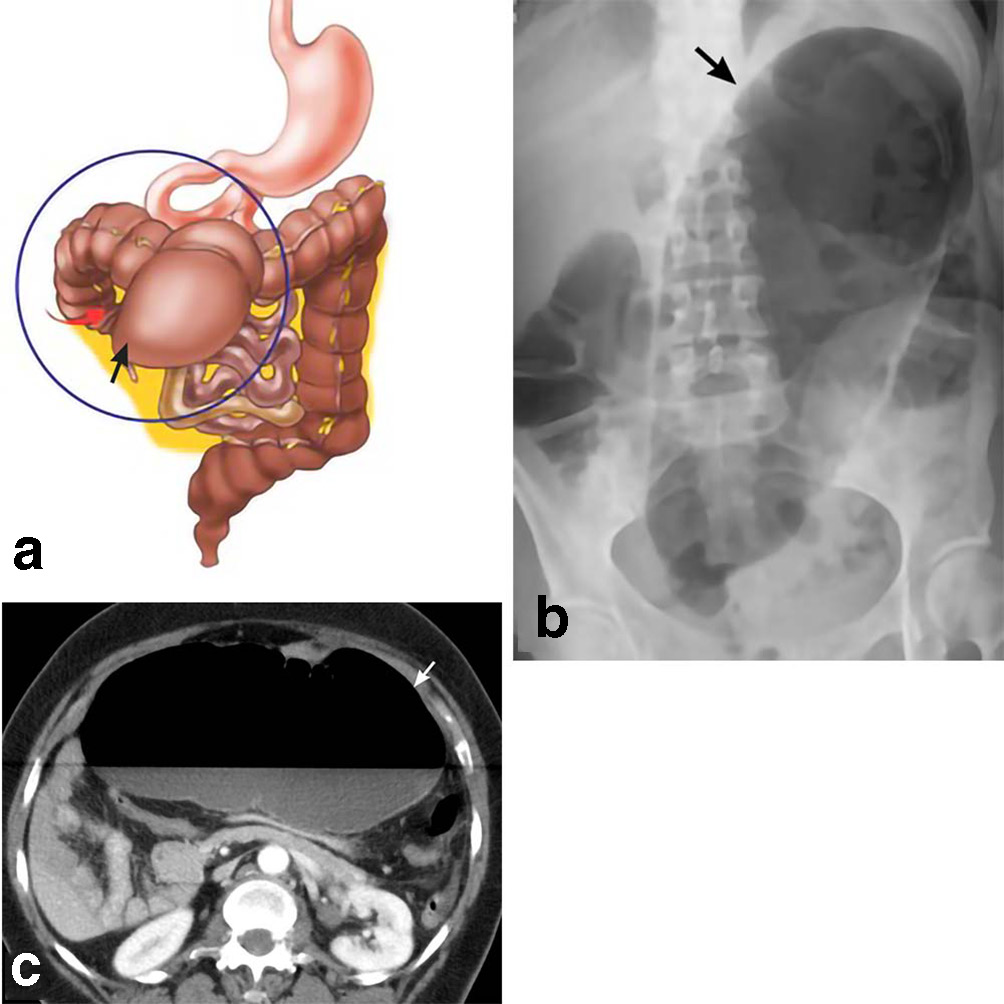
Cecal volvulus. A schematic drawing (a) depicts twisting of the cecum (red arrow) leading to cecal obstruction and dilation (black arrow). The blind ending cecum is migrating to the left upper quadrant. A 47-year-old female presents with abdominal pain. AP abdominal radiograph (b) shows a markedly dilated bowel loop extending obliquely from the pelvis towards the left upper quadrant. This represents a severely dilated cecum (black arrow) following torsion of the bowel around its mesentery. Axial CT (c) shows a massively dilated cecum (white arrow) with an air-fluid level. AP, anteroposterior.

While not specific, conventional radiography can show cecal dilatation with a single air-fluid level, small bowel dilatation, and absence of gas in the colon distal to the volvulized cecum. Barium enema characteristically shows a “beak sign” caused by the smooth tapering off of the bowel at the point of obstruction. The commonly described CT signs are the “coffee bean sign” (dilated cecum filled with air and fluid), the “whirl sign” (spiraling configuration of the fatty mesentery and dilated mesenteric vessels) and the “beak sign” seen with barium enema.^6^ Recognition of the CT signs and early diagnosis are important due to the potentially deadly outcomes of vascular compromise and bowel perforation (Figure 2).^5^

#### Cecal bascule

Cecal bascule occurs in about 10% of cases of cecal volvulus. Cecal bascule is a form of cecal volvulus in which the cecum folds ventrally over the right colon. Luminal narrowing formed at the apex of the bend obstructs emptying from the cecum.^7^

**Figure 3.**
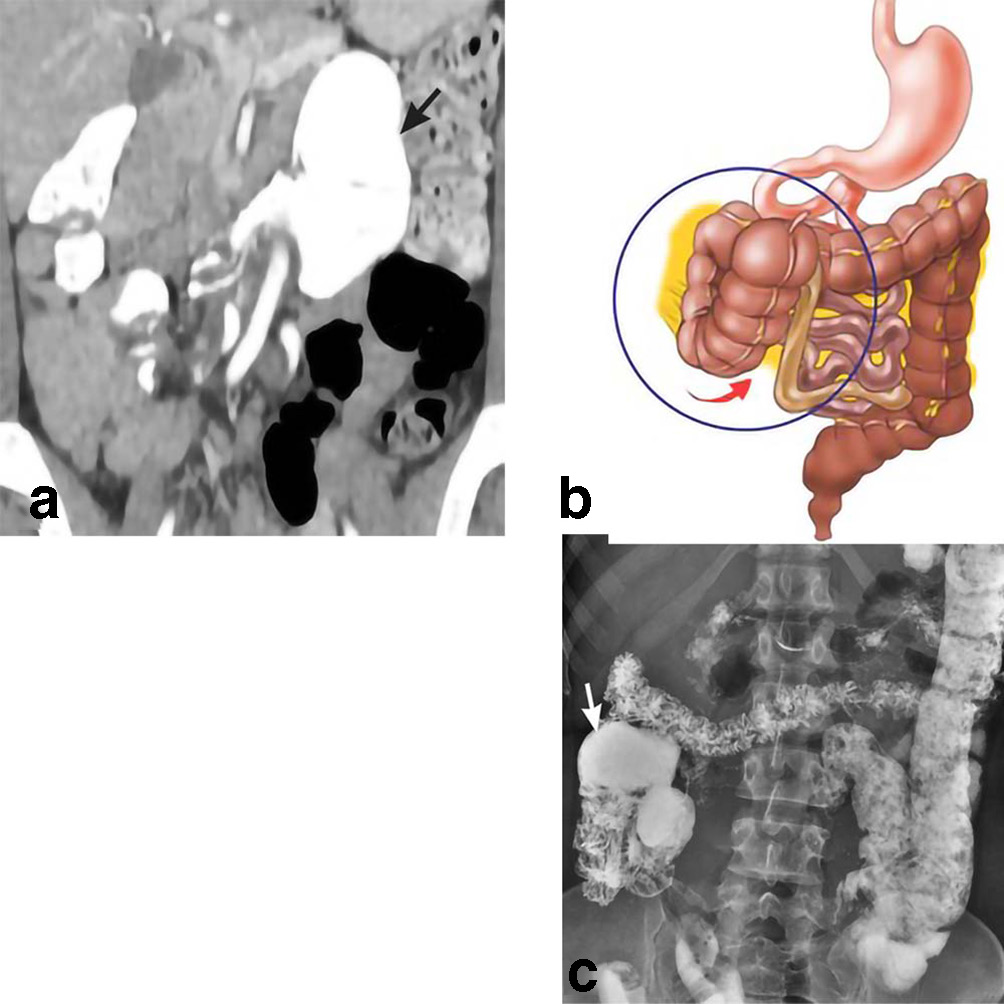
Cecal bascule. 43-year-old female presents with epigastric pain. Initial CT coronal MPR (a) shows anterior folding of the cecum to the left upper quadrant (black arrow). The drawing (b) demonstrates the ventral direction of cecal movement (red arrow). Follow-up barium enema (c) demonstrates partial reduction of cecal migration with return to the right hemiabdomen (white arrow). MPR, multiplanar reconstruction.

On imaging studies, cecal bascule often presents as a dilated loop in the mid abdomen.^5^ The presence of a constricting band across the ascending colon is a feature often found during surgery (Figure 3). Cecopexy is a surgical option in individuals who do not have ischemia.^7^

#### Ileocolonic intussusception

Ileocolonic intussusception is the invagination of the ileum with associated mesentery into the lumen of the ascending colon. This leads to impaired peristalsis, obstruction, and ischemia. Intussusception can occur without a lead point, *i.e*. primary intussusception, or with a lead point, *i.e*. secondary intussusception. Lead points include intraluminal and extraluminal lesions. While adults are less likely to have intussusception in general, when they do, they are more likely to have secondary intussusception. Clinical symptoms are generally neither sensitive nor specific enough to make a diagnosis of intussusception and therefore radiologic evidence is required.^8^

**Figure 4.**
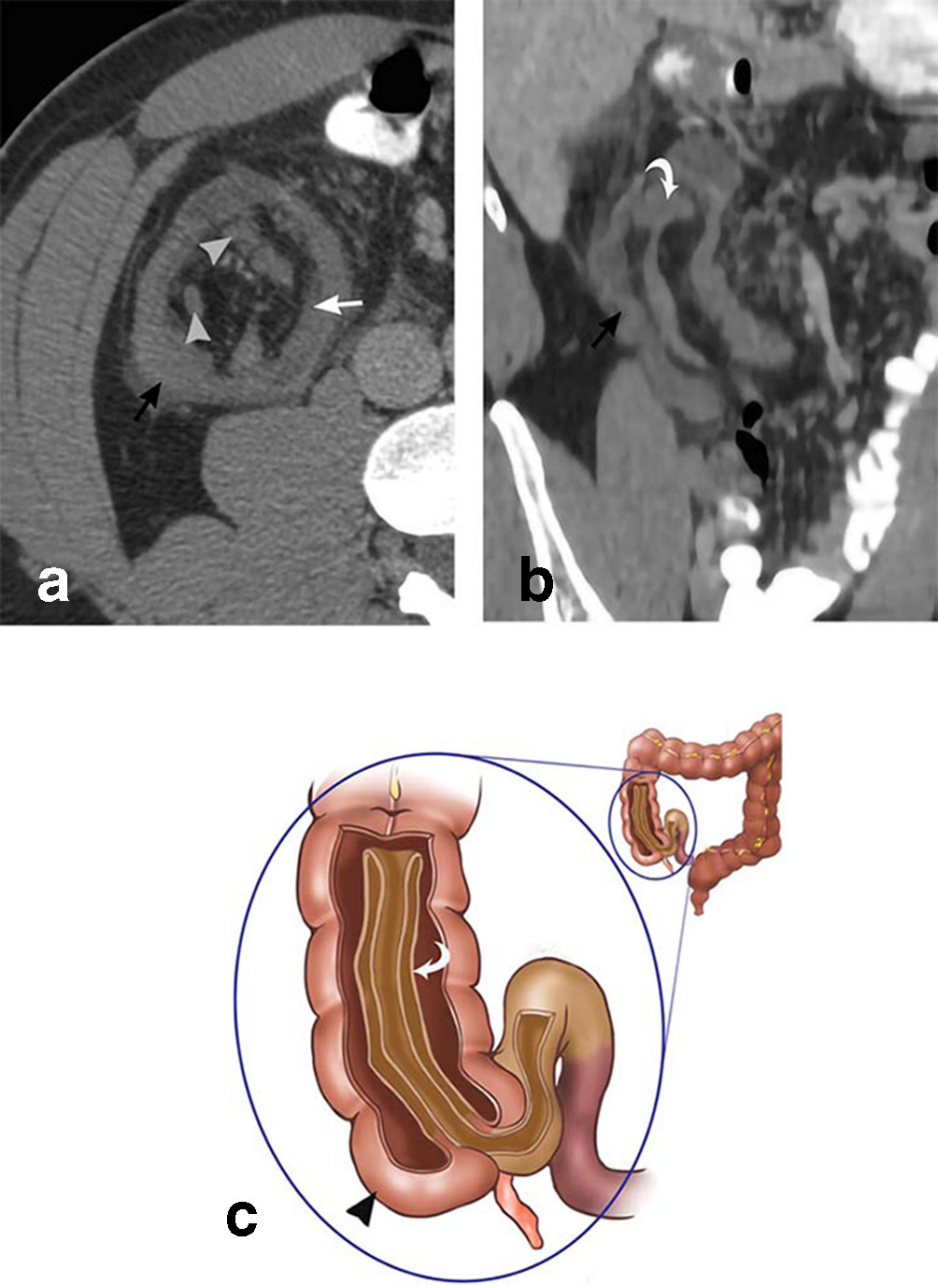
Ileocolonic intussusception. 33-year-old male presents with 1 month of vague abdominal pain. Axial CT (a) and sagittal MPR CT (b) depicts intussusception of the terminal ileum (curved white arrow), mesenteric fat (straight white arrow), and lymph nodes (gray arrowheads) into the ascending colon (black arrows). The drawing (c) depicts similar findings, with the terminal ileum (curved white arrow) telescoping into the cecum (black arrowhead). MPR, multiplanar reconstruction.

Plain radiographs may demonstrate signs of bowel obstruction or perforation, but this is not very sensitive or specific for the disease etiology. Sonography, which is more sensitive and specific, shows characteristic “target” lesions on transverse view caused by the bowel invagination. CT, the more commonly used imaging study, also shows a “target” sign and identifies the intussusceptions segments (Figure 4). The treatment of choice in adults is surgical excision of the involved bowel segments since the lead point can harbor malignancy.^8^

### Infectious and inflammatory entities of the cecum

#### Ulcerative colitis

UC is a disease characterized by mucosal inflammation of the various colonic segments with obligatory involvement of the rectum. This inflammatory bowel disease, in its classic form, includes the triad of intermittent bloody diarrhea, rectal urgency, and tenesmus. Extra intestinal manifestations include oral ulcers, osteoporosis, and arthritis. Treatment of UC is typically with anti-inflammatory or immunosuppressive therapy. Colectomy may be needed in cases of complicated UC.^9^

**Figure 5.**
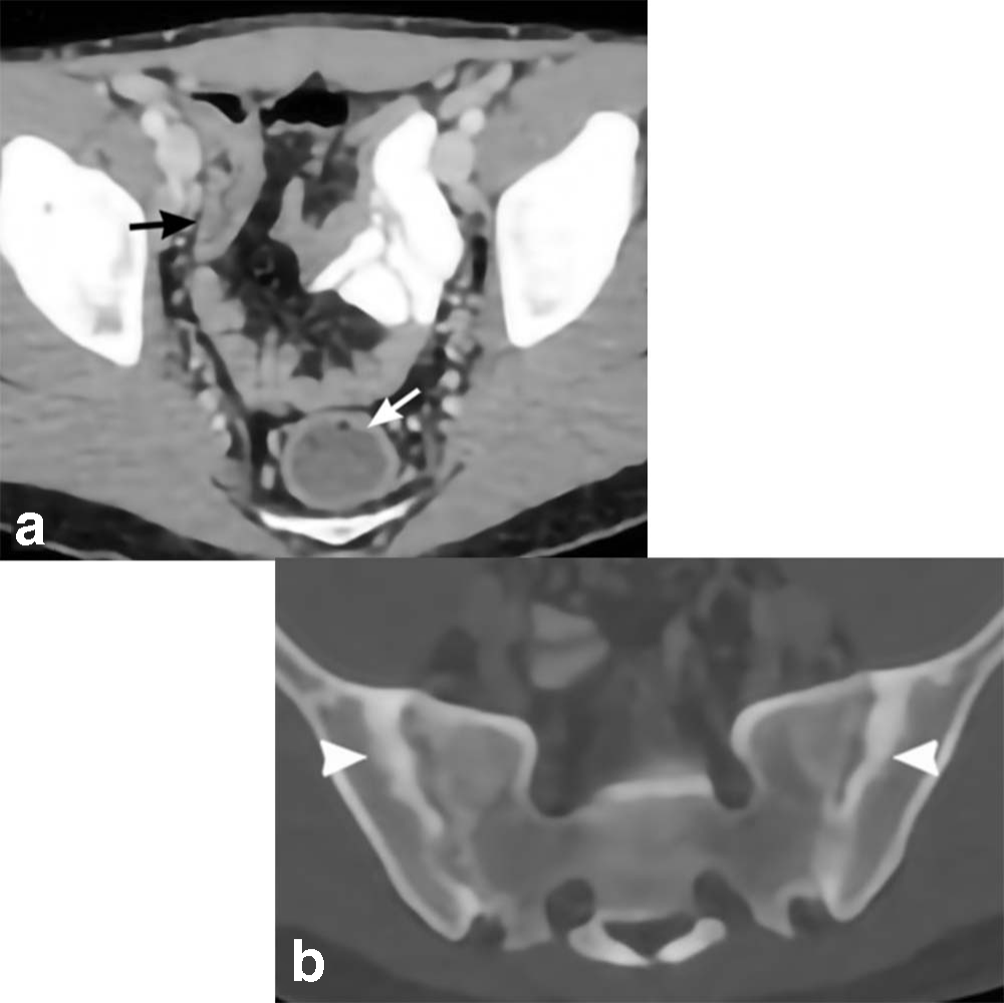
Ulcerative colitis. 24-year-old male presents with abdominal pain and chronic lower back pain. Axial CT in soft tissue windows (a) shows mild diffuse large bowel wall thickening extending from the rectum (white arrow) to the cecum (black arrow). Axial CT in bone windows (b) demonstrates bilateral sacroiliac joint sclerosis and erosions (white arrowheads) consistent with sacroiliitis, a possible extraintestinal manifestation of UC. Biopsy shows chronic moderately active colitis with cryptitis and lymphoid hyperplasia. UC, ulcerative colitis.

While not first line, CT can be used in patients with an uncertain diagnosis, to determine disease severity, and to discover extra intestinal findings. Characteristic CT findings include wall thickening, mucosal hyperenhancement, mural stratification, and pericolonic stranding (Figure 5).^10^

#### Crohn’s disease

**Figure 6.**
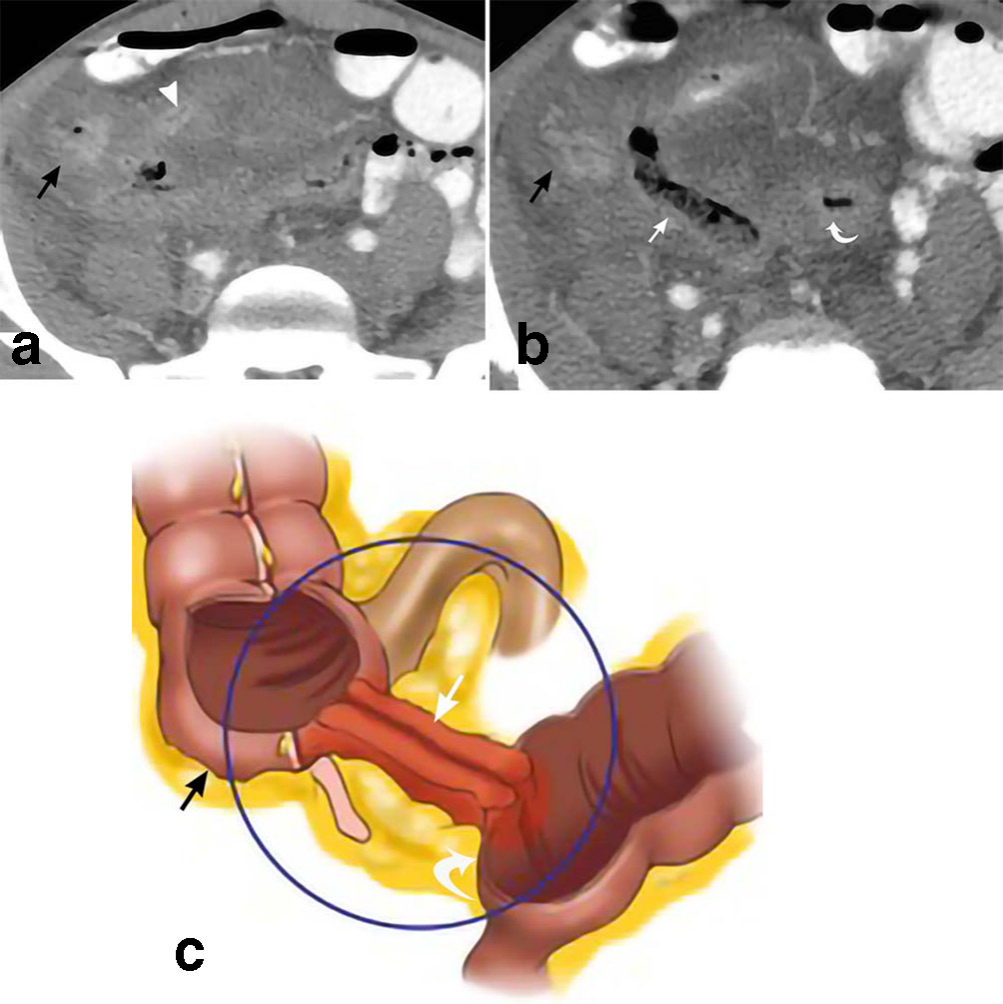
Crohn’s disease. 29-year-old male presents with three episodes of near syncope, mild intermittent abdominal pain, and 25 lbs. weight loss in 1 year. Axial CT (a) demonstrates marked wall thickening of the terminal ileum (white arrowhead) and cecum (black arrows) in a patient with Crohn’s disease. More caudal axial CT (b) depicts a fistulous tract (straight white arrow) between the sigmoid colon (curved white arrow) and cecum (black arrow). The drawing (c) depicts similar findings with a fistulous tract (straight white arrow) between the cecum (black arrow) and sigmoid colon (curved white arrow).

Crohn’s disease (CD) is a non-continuous inflammation of the intestine. Unlike UC, it usually involves the small intestine rather than the rectum. In comparison to UC, it causes more abdominal pain and less diarrhea, especially bloody diarrhea. It is important to differentiate UC and CD because they have different complications. CD is characterized by transmural inflammation leading to the creation of fistulas and sinus tracts.^9^ CT manifestations of CD can show these fistulas as well as bowel wall thickening that tends to be thicker than in UC (11 *vs* 8 mm) (Figure 6).^10^ MR enteropathy can be used to diagnose and demonstrate the severity of CD. Classic findings on MR enteropathy includes mural thickening, mural hyperenhancement, and creeping fat with mural hyperenhancement being the most sensitive finding.^11^

#### Inflammatory changes secondary to acute appendicitis

**Figure 7.**
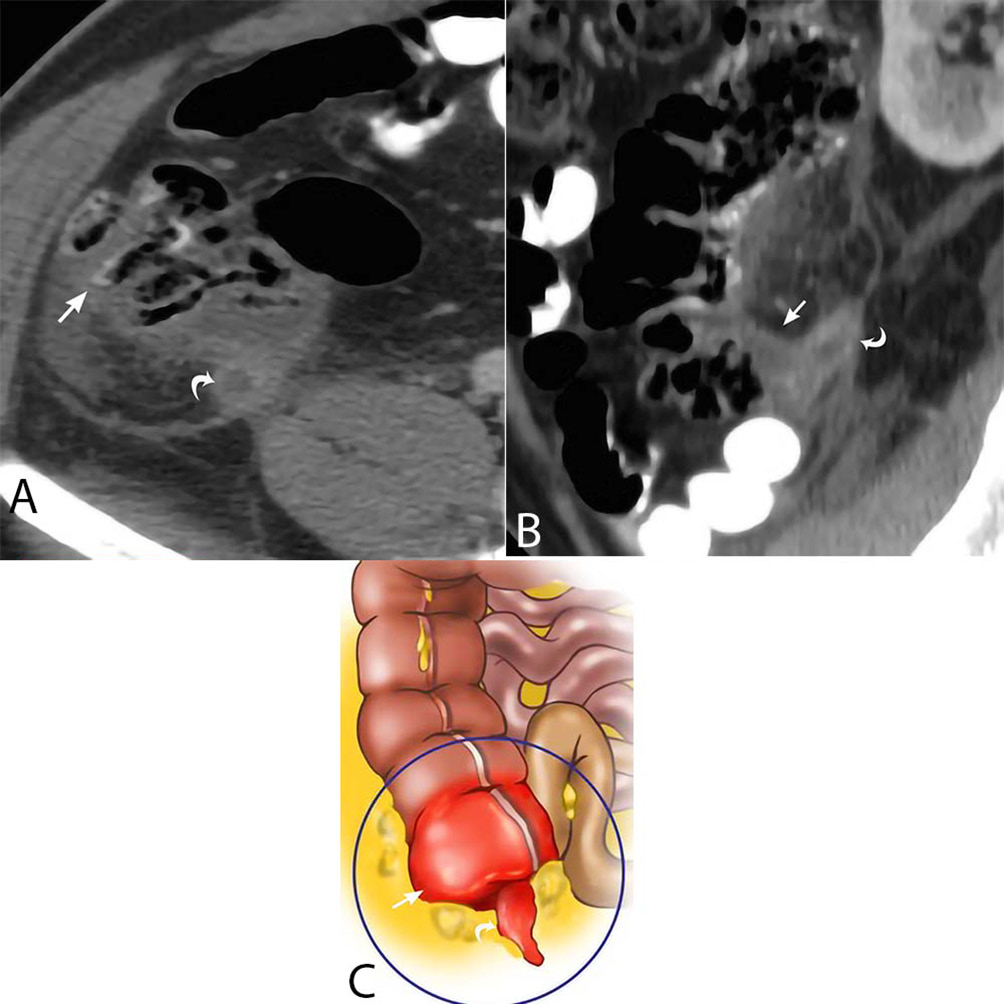
Inflammatory changes secondary to acute appendicitis. 42-year-old male presents with mid-abdominal pain, nausea and vomiting for 2 days. Axial CT (A) and sagittal MPR CT (B) depict the enlarged appendix (curved white arrows) with surrounding inflammatory soft tissue stranding and secondary inflammatory changes of the cecum, including cecal wall thickening (straight white arrows). The drawing (C) shows similar findings. MPR, multiplanar reconstruction.

In acute appendicitis, CT shows a dilated appendix with a diameter greater than 6 mm. Other characteristic signs include periappendiceal inflammation, mucosal enhancement, and thickening of the appendiceal wall, which can extend into the cecum and terminal ileum. A thickened inflamed cecum can form an arrowhead-shaped collection of contrast pointing to the occluded appendix. This is a secondary sign of acute appendicitis (Figure 7).^12^

#### Cecal diverticulitis

Diverticulosis is very common in the Western world with approximately 50% of people over the age of 50 having colonic diverticula. Most diverticula occur in the sigmoid, though right-sided diverticula occurs more frequently in younger patients. Cecal diverticulosis is thought to be due to a congenital prenatal lesion and is usually asymptomatic.^13^

**Figure 8.**
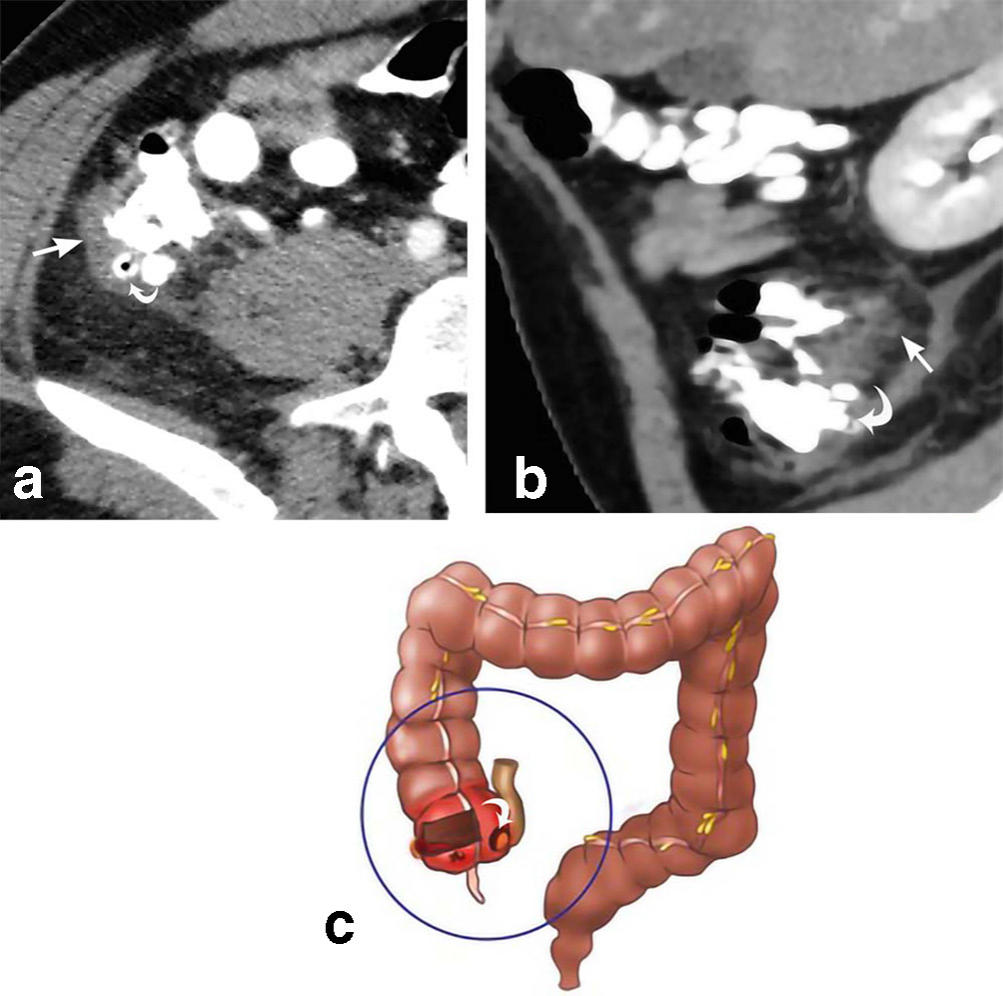
Cecal diverticulitis. 37-year-old male presents with right lower quadrant abdominal pain. Axial CT (a) and sagittal MPR CT (b) depict cecal diverticula (curved white arrows) with surrounding inflammatory soft tissue stranding. Cecal wall thickening (straight white arrows) is also demonstrated. The drawing (c) depicts the same. MPR, multiplanar reconstruction.

While ultrasound can be used in right-sided diverticulitis, CT is more sensitive. CT findings in cecal diverticulitis, similar to the left-sided diverticulitis, include the presence of diverticula, bowel wall thickening, focal soft tissue stranding, thickening of the regional facial planes, and mass effect by the enlarged inflamed diverticulum.^13^ High-resolution CT scans are very sensitive and specific at diagnosing diverticultitis (Figure 8).^14^

#### *Clostridium difficile*/pseudomembranous colitis

*Clostridium difficile* (*C. difficile*) is a Gram-positive bacterium that can cause diarrhea, lower abdominal pain, and abdominal tenderness. Previous hospital admission is the most significant risk factor for this infection. Previously, *C. difficile* was thought to be exclusively due to prior antibiotic use but recent research suggests that other factors, such as immunosuppression, also play a role. *Clostridium difficile* is a key factor in the development of pseudomembranous colitis, which is defined by the presence of pseudomembranes on the colonic or small intestine mucosa. It also plays a role in the development of sepsis, toxic megacolon, and fulminant colitis. Treatment of *C. difficile* colitis requires infection control and antibiotic therapy (such as oral Vancomycin use) with the possibility of surgery in refractory cases.^15^

**Figure 9.**
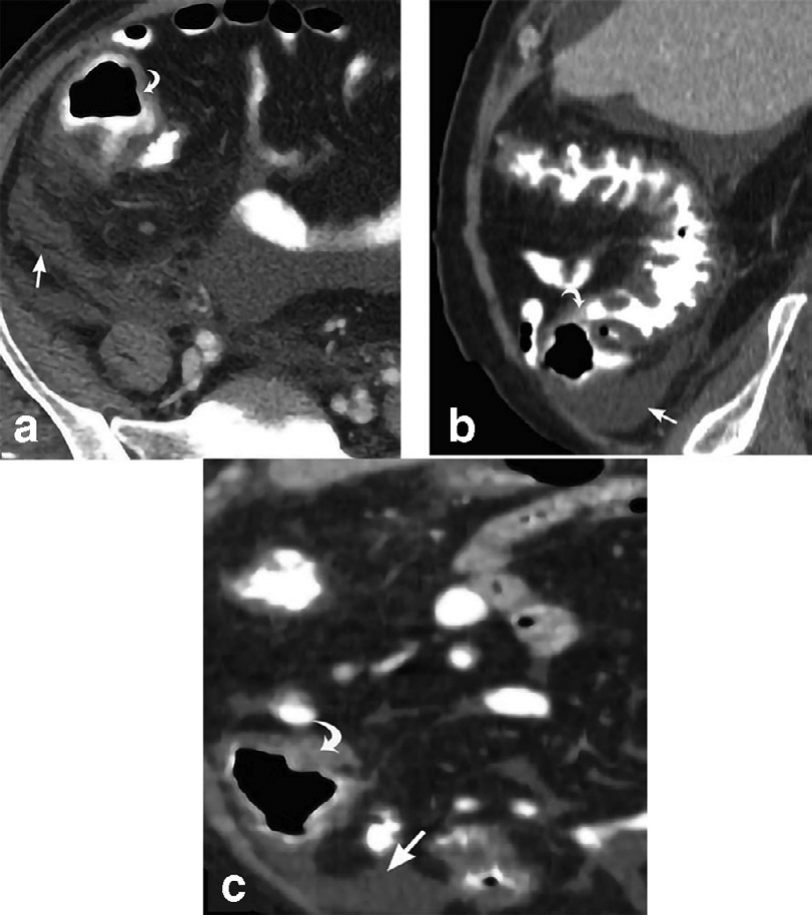
Pseudomembranous colitis. 54-year-old male presents with diarrhea for 3 days. Diffuse colonic wall thickening involving the cecum (curved white arrows) is depicted on axial CT (a) and sagittal MPR CT images (b, c). There is surrounding inflammatory soft tissue stranding and free fluid (straight white arrows). Subsequent stool assay reveals that the patient was positive for the *C. difficile* toxin. Sigmoid biopsy reveals colonic mucosa with superficial necrosis and fibrinopurulent exudate. MPR, multiplanar reconstruction.

The most common CT finding is colonic wall thickening in the range of 3–32 mm. Additional findings include the recognizable low density of the bowel wall produced by mucosal and submucosal swelling, pericolonic stranding, and ascites. While highly specific, the “accordion sign” (high density intestinal contrast alternating with the transverse edematous mucosal folds) is usually seen only in advanced disease (Figure 9).^16^

#### Cytomegalovirus (CMV) colitis

CMV is a herpes virus that, like other herpes viruses, can be transmitted by bodily fluids. CMV remains dormant after initial infection, but with impairment of cell-mediated immunity, viral reactivation can occur. Colitis is a common gastrointestinal manifestation of CMV, especially in those who are immunocompromised, such as patients with HIV/AIDS. Early diagnosis is the key to preventing disease dissemination.^17^

**Figure 10.**
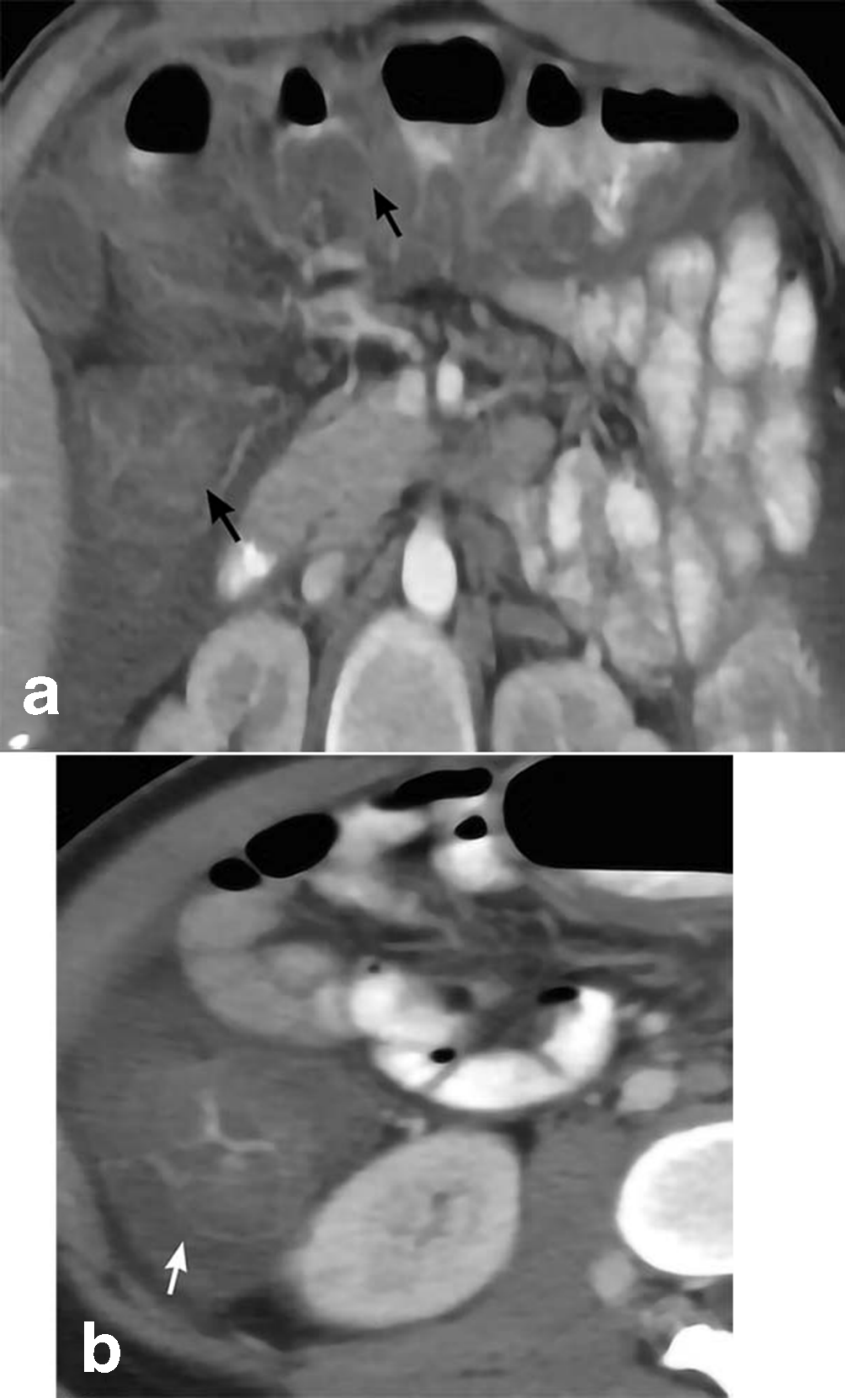
CMV colitis. 23-year-old male with medical history of HIV presents with diffuse watery diarrhea and abdominal pain for 5 days. Axial CT imaging (a) depicts markedly diffuse colonic wall thickening of the ascending and transverse colon (black arrows). The cecum is uniformly thickened (white arrow) as depicted on the axial CT (b). Subsequent sigmoid biopsy reveals colonic mucosa with ischemic changes and numerous viral inclusion cysts compatible with CMV infection. CMV, cytomegalovirus.

CMV colitis can involve the cecum, descending colon, and rectosigmoid junction. The most common CT finding in this disease is colonic wall thickening (Figure 10). Alternating mural layers of high and low attenuation have been seen in a majority of the cases. Patients with HIV/AIDS usually also have mural ulcerations. Additionally, some nonspecific findings such as ascites and pericolonic fat stranding can also be seen. Colonic perforation is a potentially fatal complication of CMV colitis especially given the fact that these patients are afebrile, present with minimal abdominal tenderness, and have a normal WBC count.^17^

#### Tuberculosis

Tuberculosis (TB) infection can affect any of the abdominal organs. TB peritonitis, a widespread form of infection involving the peritoneal and mesenteric structures, is most common. TB can also involve a focal bowel segment or present as diffuse enteritis. Most cases affect the ileocecal valve and the adjacent ileum and cecum. In these cases, the initial insult occurs at the epithelioid tubercle, which is located in the submucosal lymphatic tissue. This undergoes caseous necrosis over the next month. The ulcerations then coalesce and the adjacent mesenteric lymph nodes become involved following lymphatic spread. This leads to cecal wall thickening and causes the surrounding lymph nodes to adhere to the wall of the cecum and terminal ileum forming an inflammatory mass.^18^

**Figure 11.**
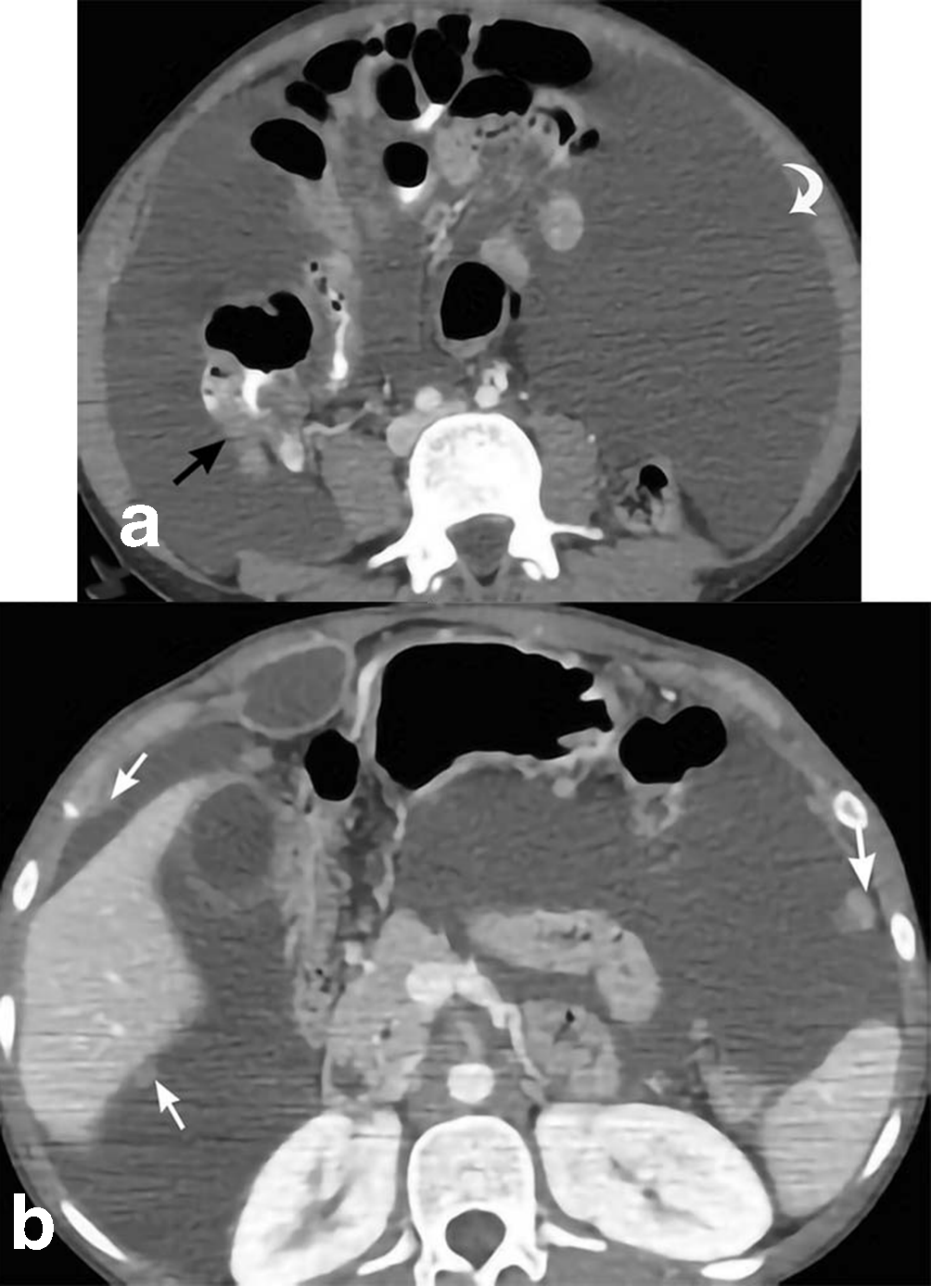
Tuberculosis. 22-year-old female presents with diffuse abdominal pain and ascites. Axial CT (a) shows massive ascites (curved white arrows) and thickening of the ascending colon including the cecum (black arrow). Axial CT (b) depicts peritoneal nodules (straight white arrows). Colonoscopy reveals colonic mucosa with severe ulceration, granulomas, and granulation tissue with positive AFB stain and culture. AFB, acid-fast bacteria.

The most characteristic appearance of TB on CT is an enlarged ileocecal valve with a thickened cecal and/or terminal ileal wall. Pericecal regional lymphadenopathy with caseous necrosis and peritoneal involvement may also be present (Figure 11). Barium study can show thickened folds, spasticity, irregular contours and superficial ulcerations. Thickening of the ileocecal valve associated with a narrowed terminal ileum can also be seen on barium examination.^18^

## Conclusion

The clinical manifestations of cecal disease can be diverse and misleading. There is also great overlap in clinical presentations. Thus, the radiologist may the first to diagnose these clinically ambiguous entities. Mastery of the broad spectrum of cecal pathologies and their respective imaging appearances on CT can be invaluable in helping radiologists define the diagnosis and recommend appropriate treatment management.
